# Supplementary figures and images for: Dating Whole Genome Duplication in Ceratopteris thalictroides and Potential Adaptive Values of Retained Gene Duplicates
Source: Int J Mol Sci. 2019 Apr 19;20(8):1926. doi: 10.3390/ijms20081926 (PMC6515051; doi:10.3390/ijms20081926)

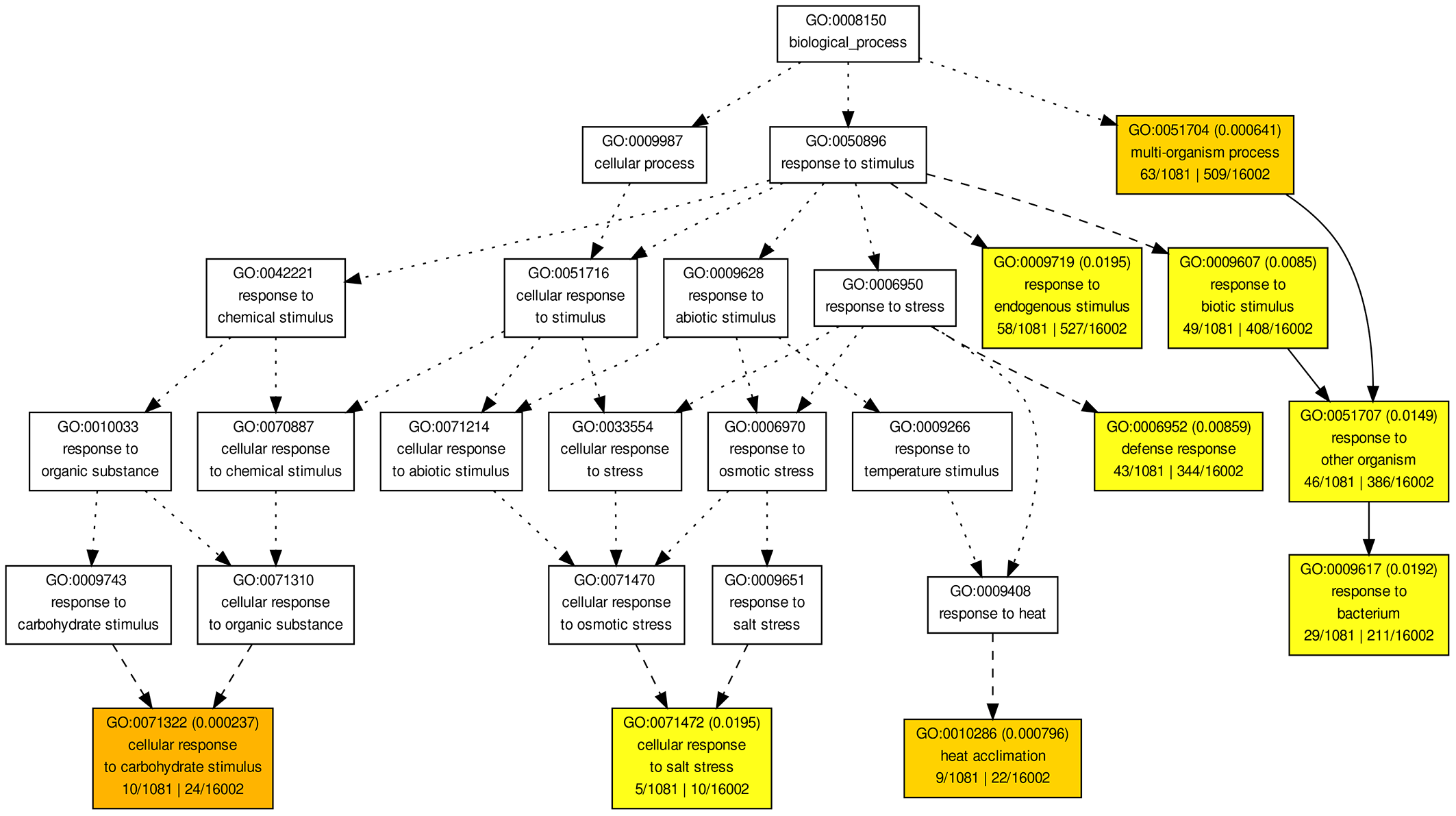

Supplement: Supplementary file 1 [file ijms-20-01926-s001.zip › ijms-474761-supplementary/Figure S1 Enriched GO terms of retained duplicates related to environmental response after WGD events in the diploid of C. thalictroides.tif]

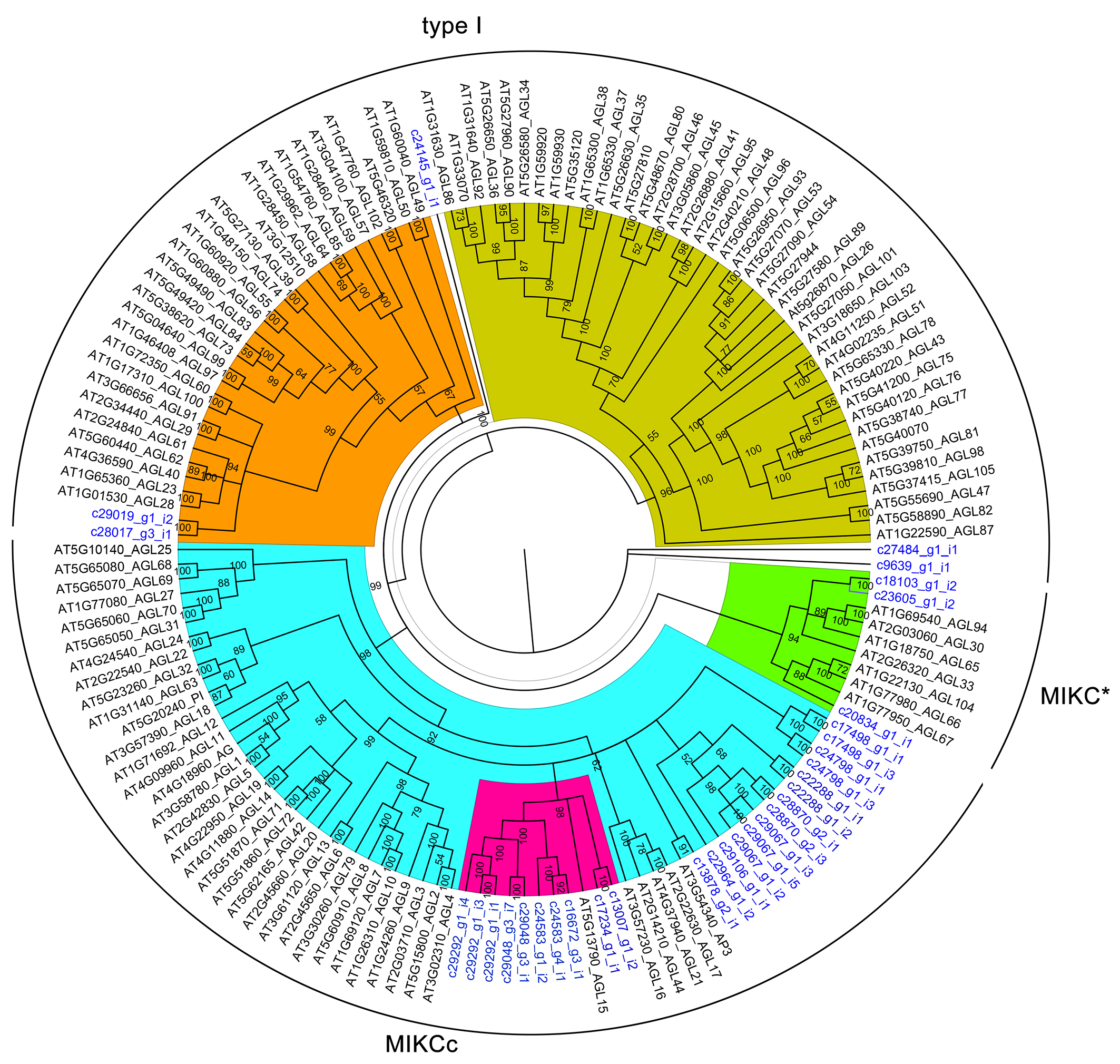

Supplement: Supplementary file 1 [file ijms-20-01926-s001.zip › ijms-474761-supplementary/Figure S2 Phylogenetic tree of MADS-box genes in A. thaliana, and C. thalictroides. Gene names of C. thalictroides are colored with blue. And posterior probabilities are indicated on the branches.TIF]

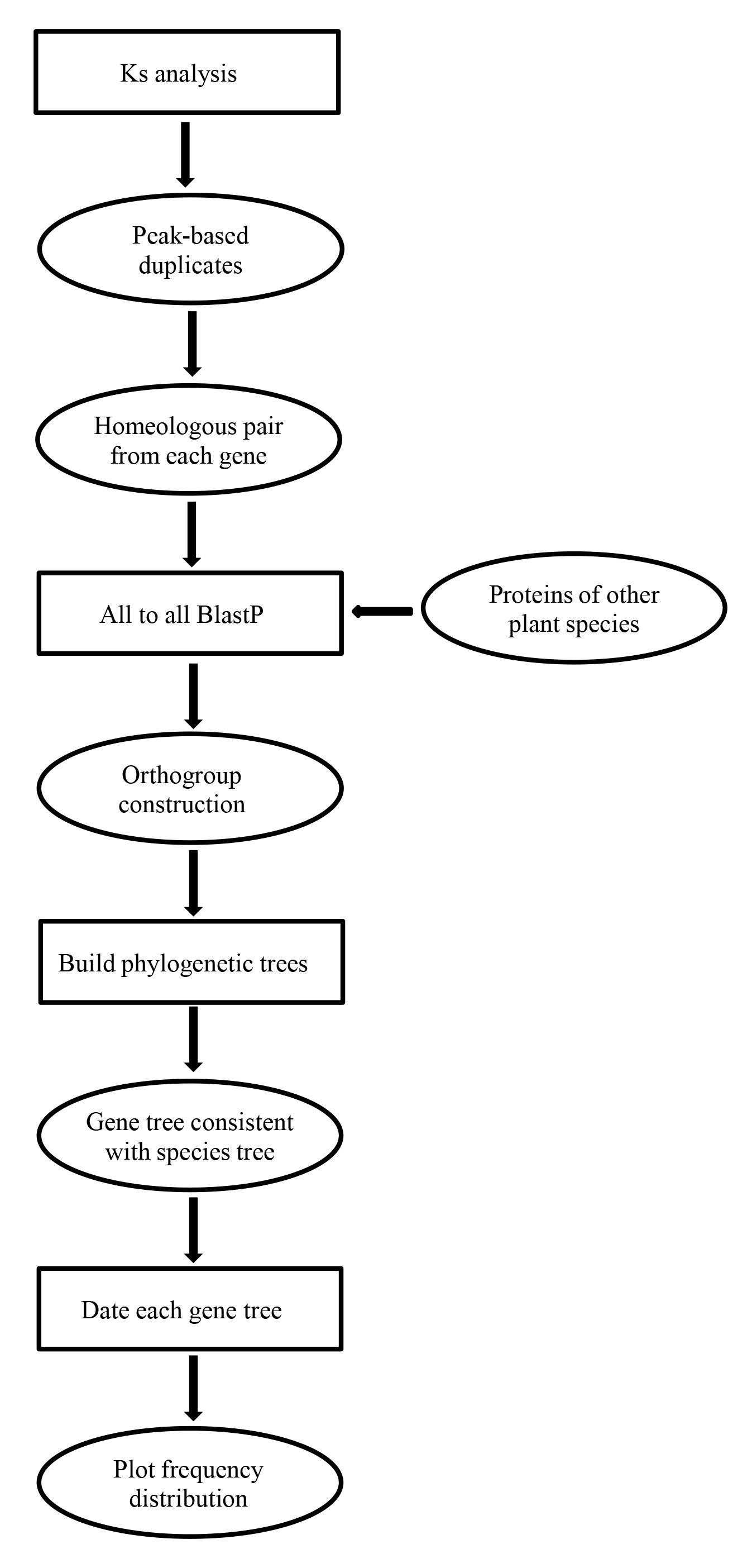

Supplement: Supplementary file 1 [file ijms-20-01926-s001.zip › ijms-474761-supplementary/Figure S3 A work flow diagram showing the major processoes of aboslolute dating analysis in this study.JPEG]

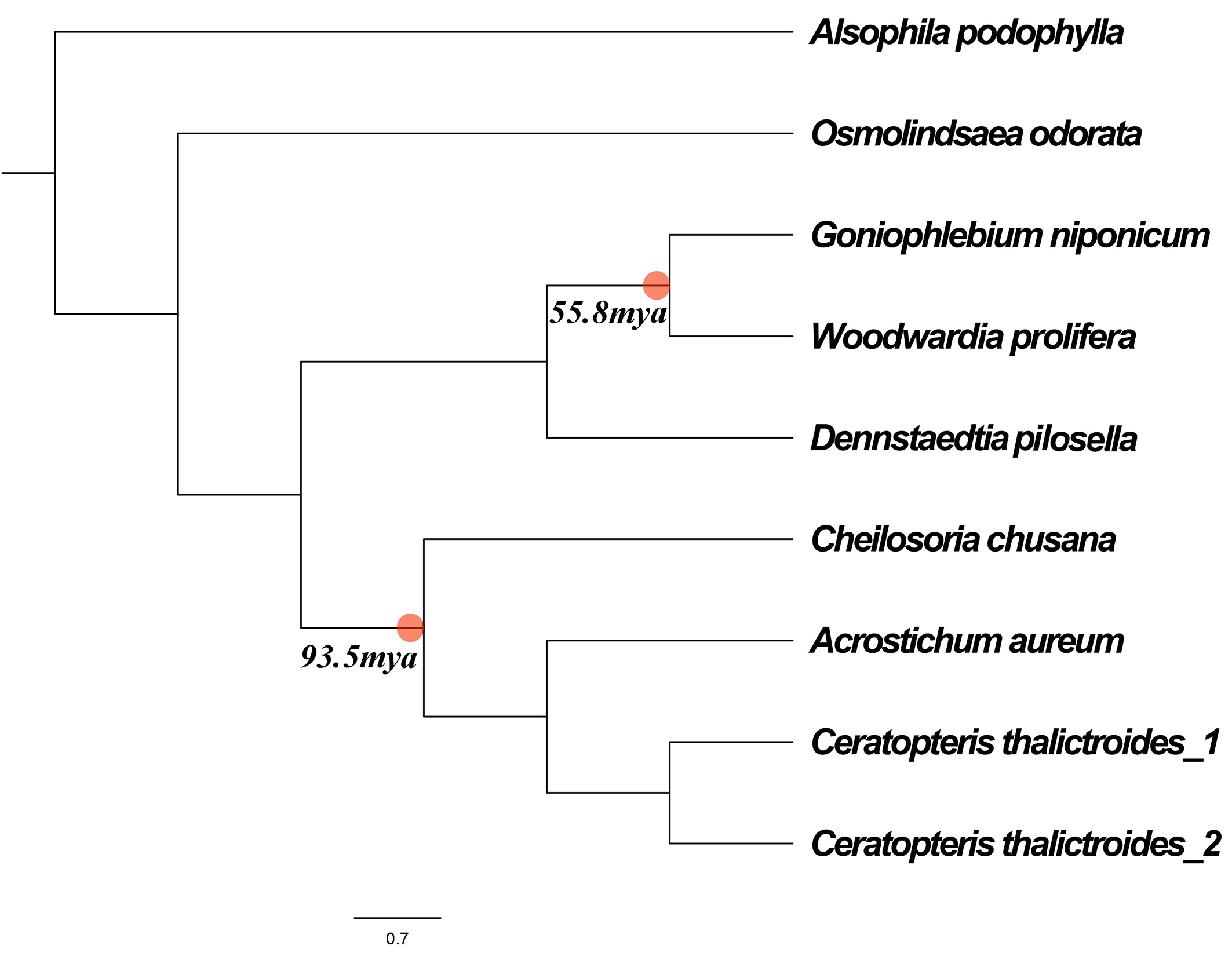

Supplement: Supplementary file 1 [file ijms-20-01926-s001.zip › ijms-474761-supplementary/Figure S4 Gene tree topology for an orthogroup.jpg]
